# Effects of Micro- and Nanosilica on the Mechanical and Microstructural Characteristics of Some Special Mortars Made with Recycled Concrete Aggregates

**DOI:** 10.3390/ma17122791

**Published:** 2024-06-07

**Authors:** Claudiu Mazilu, Radu Deju, Dan Paul Georgescu, Adelina Apostu, Alin Barbu

**Affiliations:** 1 Roads, Railways and Construction Materials Department, Faculty of Railways, Roads and Bridges, Technical University of Civil Engineering, 020396 Bucharest, Romania; 2 Horia Hulubei National Institute for Physics and Nuclear Engineering, P.O. Box MG-6, Măgurele, 077125 Bucharest, Romania; 3 Reinforced Concrete Construction Department, Faculty of Civil and Industrial Constructions, Technical University of Civil Engineering, 020396 Bucharest, Romania; 4 Doctoral School, Reinforced Concrete Structures, Technical University of Civil Engineering, 020396 Bucharest, Romania

**Keywords:** special mortars, microsilica, colloidal nanosilica, recycled concrete aggregates, physical–mechanical properties, microstructure

## Abstract

In this paper, we study the influence of densified microsilica and colloidal nanosilica admixtures on the mechanical strength and the microstructural characteristics of special mortars used for immobilizing radioactive concrete waste. The experimental program focused on the replacement of cement with micro- and/or nanosilica, in different proportions, in the basic composition of a mortar made with recycled aggregates. The technical criteria imposed for such cementitious systems, used for the encapsulation of low-level radioactive waste, imply high fluidity, increased mechanical strength and lack of segregation and of bleeding. We aimed to increase the structural compactness of the mortars by adding micro- and nanosilica, all the while maintaining the technical criteria imposed, to obtain a cement matrix with high durability and increased capacity for immobilizing radionuclides. The samples from all the compositions obtained were analyzed from the point of view of mechanical strength. Also, micro- and nanosilica as well as samples of the optimal mortar compositions were analyzed physically and microstructurally. Experimental data showed that the mortar samples present maximum compressive strength for a content between 6 and 7.5% wt. of microsilica, respectively, for a content of 2.25% wt. nanosilica. The obtained results suggest a synergistic effect of micro- and nanosilica when they are used simultaneously in cementitious compositions. Thus, among the analyzed compositional variants, the mortar composition with 3% wt. microsilica and 2.25% wt. nanosilica showed the best performance, with an increase in compressive strength of 23.5% compared to the control sample (without micro- and nanosilica). Brunauer–Emmett–Teller (BET) analysis and scanning electron microscopy (SEM) images highlighted the decrease in pore diameter and the increase in structural compactness, especially for mortar samples with nanosilica content or a mixture of micro- and nanosilica. This study is useful in the field of recycling radioactive concrete resulting from the decommissioning of nuclear research or nuclear power reactors.

## 1. Introduction

Microsilica (MS), due to its high-purity SiO_2_ particles with dimensions up to 500 nm, has become, over time, one of the most used supplementary cementitious materials for improving the durability of mortars and concretes [1]. The performance increase in cement systems has passed a critical point with the use of superfine particles that appeared due to the development of nanotechnology. Obtained by various methods such as sol–gel, vaporization, precipitation and biological methods [2], nanosilica (NS) is currently a nanomaterial often used in mortars and concretes to improve the physico-chemical and mechanical properties. Due to the very small size of the SiO_2_ particles (<100 nm), both the pozzolanic activity and the microfiller effect are more pronounced in the case of nanosilica compared to microsilica, resulting in a compact, dense and homogeneous microstructure [3] that leads to an increase in mechanical resistance for cement systems [4]. Also, the reduction in the pore size and the interruption of structural capillarity leads to a reduction in the water permeability simultaneously with the increase in chemical resistance [5]. Nanosilica assists in cement hydration in an early phase [6] through the nucleation effect of the nanoparticles, which decreases the setting time and increases the hardening velocity. Furthermore, more studies [7,8] have shown that NS accelerates tricalcium silicate hydration and intensifies the development of calcium hydrosilicates (CSHs) with gel structures.

Nanosilica can be used either as a dry powder or in the colloidal form [9]. Amorphous SiO_2_ particles from colloidal nanosilica present better dispersion in the cement matrix and are considered to facilitate the production of CSH gel with high stiffness [10]. Recent studies [11,12] have shown that nanosilica should not be added alone to the composition of mortars and concretes, but together with microsilica to successively fill the voids and increase the microstructural density of hardened cementitious systems [13]. It was established that the addition of only 1% NS has the same effect as the use of 10% MS and the combined addition of micro- and nanosilica offers a synergistic effect [14], in the sense that it gives better results than the sum of the individual effects.

In recent decades, with the worsening of environmental problems related to pollution and the conservation of natural resources, it has been considered a viable solution to replace natural aggregates from the composition of cement systems with recycled aggregates obtained by crushing concrete waste [15]. Earlier works [16,17] confirmed that cement systems using recycled aggregates had some inferior properties, like low density, more water absorption and reductions in durability, due to the mortar that remains attached to natural aggregate (NA) [18,19]. Along with mixing techniques, the addition of supplementary cementitious materials, such as micro- and nanosilica, is another significant method of improvement for properties of mortars and concretes with recycled aggregates [20]. The mechanisms by which micro- and nanosilica act in cement systems with recycled aggregates are similar to those presented in the case of using natural aggregates. The difference in the case of using recycled aggregates is that micro- and nanosilica act mainly in the interfacial transition zone (ITZ) around the aggregate granules by densifying and reducing the porosity of the old mortar attached to the natural aggregate [21]. One cement system made with recycled aggregates is represented by special mortars for encapsulating low-level radioactive waste (LLW).

The concrete waste resulting from the decommissioning of nuclear installations is mostly low-level radioactive waste [22,23]. The final storage of these materials causes significant problems because the sites and techniques for safe final storage of radioactive waste are limited and expensive [23]. Reducing the waste storage volume is essential for reducing the cost of decommissioning nuclear installations. Currently, this type of waste is placed in cylindrical steel containers and solidified with mortar prepared with natural aggregates [24,25,26].

In the case of using natural aggregates to obtain the encapsulation mortar, a degree of filling of about 50% vol. of the concrete LLW waste containers is realized [26]. Recent studies [27,28,29] have shown that the increase in the degree of filling of the containers with radioactive waste up to about 75% vol. was achieved by recycling the radioactive concrete and using it as a fine fraction in the composition of the encapsulation mortar.

Mortar used for embedding low-level concrete waste (LLW) must meet specifications applicable to LLW waste. The technical criteria for LLW waste stipulated in Article 8 of the Ordinance of the Ministry of Economy, Trade and Industry of Japan “Waste Burial Rules” were taken as targets [25,28].

In fresh conditions, according to the rheological properties, the LLW encapsulation mortar requires an optimal flow (in the range of 16–35 s/1000 mL mortar) through a Marsh funnel with a nozzle diameter of 10 mm, to ensure a maximum degree of filling of the waste container [28]. Likewise, the mortar in its fresh state must not show segregation or separate water over time (bleeding). The fluidity and cohesion of the fresh mortar can be improved both by using additives and/or mineral additions, as well as by adjusting the water content. The compositional changes are made so as to ensure, in a hardened state, a compression resistance of the encapsulation mortar of at least 30 MPa as well as its reduced porosity and permeability [28].

A solution for improving the structural compactness of LLW encapsulation mortars and limiting the leaching of radionuclides from the composition consists of the use of various mineral additions that ensure an increased immobilization of radionuclides in the cementoid matrix [30,31].

This work seeks to improve the composition of the LLW encapsulation mortar made with recycled aggregates in the framework of previous research [27] by adding micro- and/or nanosilica in different percentages and choosing the optimal variant from a compositional point of view so as to increase the capacity of encapsulation and radiological protection. The separate and simultaneous influence of the content of densified microsilica and colloidal nanosilica on the properties in the fresh and hardened states of the mortars will be analyzed compared to the control composition without silica. The compositional optimization considers an analysis of the values of the mechanical strength and the microstructure of the mortar samples with micro/nanosilica content, under the conditions of keeping the technical specifications mentioned for such special cementoid systems.

The novelty of this study consists of the use of micro- and nanosilica to increase the performance of some mortars with special technical characteristics, made with recycled concrete aggregates. In the specialized literature, there is information regarding solidification/stabilization technology for radioactive waste [22,23], based on Portland cement with supplementary cementitious materials (SCMs) [32]; they do not refer to cement systems made with recycled concrete aggregates but to those obtained with natural aggregates.

Such special mortars used for encapsulating low-level radioactive waste (LLW) have applicability in the recycling of radioactive concrete resulting from the decommissioning of nuclear installations. Decommissioning projects of nuclear installations from other countries of the world and from Romania are considered, such as the TRIGA-type research reactor at the Nuclear Research Institute in Pitesti-Mioveni (after 2030) and the nuclear–electric reactors in Cernavoda (after 2050).

## 2. Materials and Methods

The studied mortars have the following composition: cement, recycled concrete aggregates (particle size fraction 0–5 mm), superplasticizer additive, viscosity modifier additive, water and cement replacement admixture: densified microsilica (MS), colloidal nanosilica (CB) or a mixture of these two.

CEM I 42.5 R Portland cement, manufactured by Heidelbergcement Fieni, Fieni, Romania, was used to prepare the mortars. The chemical composition for cement and microsilica was determined by elemental chemical analysis using a sequential X-ray fluorescence (XRF) spectrometer with wavelength dispersion, type S8 Tiger, Bruker–AXS, Berlin, Germany.

The specific BET surface area, the average diameter of the open pores and the total pore volume for cement, microsilica and mortars were determined using a Autosorb-1C device, Quantachrome Instruments, Gainesville, FL, USA.

The recycled concrete aggregates (RCAs) came from a batch of C20/25 class concrete, crushed in 2 stages (B1624J Schutte-Buffalo jaw crusher and WA-12 H Schutte-Buffalo hammer crusher) [33,34], in accordance with the decommissioning technology of nuclear installations developed and presented previously [27,33]. Recycled concrete aggregates, with a range in particle sizes of 0–5 mm, were used to obtain the mortars proposed in the present study.

The superplasticizer additive used was a strong water-reducing additive obtained on the basis of polycarboxylate ether, and together with the viscosity modifier was purchased from the BASF Romania company (from Bucharest). Both additives were used according to the technical specifications. In addition, the compatibility between the cement, superplasticizing additive and the micro/nanosilica was determined by measuring the flow time of a paste of standard consistency (determined volume) through a Marsh funnel provided with a 10 mm nozzle.

Colloidal nanosilica (CB), type Levasil CemBinder8, represents an aqueous alkaline dispersion of amorphous SiO_2_ nanoparticles (≈50%wt. SiO_2_ content) with a negatively charged surface, stabilized with sodium. A series of physico-chemical characteristics of the product are presented by the manufacturer (Nouryon from Bohus, Sweden) in the technical sheet [35].

In the cementitious matrices, the small and spherical particles of amorphous SiO_2_ from the colloidal dispersion react with Ca(OH)_2_ resulting from the hydration–hydrolysis reactions of the cement forming additional amounts of calcium hydrosilicates (CSHs) that densify and strengthen the structure [3,4,12,36,37,38,39,40,41].

Because the dispersion medium of colloidal nanosilica is water, the pH was determined in the present study with the help of a Hanna bench pH/ISE meter HI 4222 pH-meter (manufactured by Hanna Instruments from Woonsocket, RI, USA), and the density of the colloidal system was determined with the help of a pycnometer.

For densified microsilica (MS) in the form of powder, type MasterRoc MS 610 (from BASF Bucharest, Romania), a series of physico-chemical characteristics were also measured, such as relative humidity, loss of calcination, bulk and relative density, etc. The particle size distribution of microsilica (MS) was determined by sieving 200 g of powder with a Retsch sieve for 2 min, using a sieve with a diameter of 200 mm and a mesh size of 0.009; 0.125; 0.250; 0.500; and 1.0 mm.

Samples of microsilica (MS) and mortars with micro/nanosilica were subjected to microstructural analysis using a field emission scanning electron microscope with integrated focused ion beam (FESEM-FIB), coupled with an energy dispersive spectroscope (EDS) mounted on a microscope, for elemental chemical point analysis (manufactured by Hitachi Ltd. from Tokyo, Japan).

The Chapelle method, applied according to the provisions of the French standard NF P 18-513 [42,43,44], was used to assess the pozzolanic activity of densified microsilica and nanosilica. The method involves measuring the content of Ca(OH)_2_ remaining after combining it with SiO_2_. The consumed quantity of CaO or Ca(OH)_2_ must be greater than 660 mg CaO (871 mg Ca(OH)_2_)/1 g of silica for it to be considered pozzolanic material.

The amount of Ca(OH)_2_ consumed (linked to silica) was determined with the following relation:

mgCa(OH)_2_ = 2·[(v_2_ − v_1_)/v_2_] × 74/56 × 1000 (1)

where the variables represent the following:

v_2_ represents the volume of HCl solution, 0.1 N, used for the titration of the control mixture (CaO);
v_1_ represents the volume of HCl solution, 0.1 N, used for the titration of the pozzolanic mixture (CaO + micro/nanosilica).

### 2.1. Compositions

In the present study, LLW encapsulation cement systems were prepared starting from the composition of basic mortars based on earlier research and obtained with recycled aggregates [27].

The additions of micro- and nanosilica were incorporated in the mortar compositions in the following proportions:

- For densified microsilica (MS), 3% wt. (composition noted as MS3); 4.5% wt. (MS4.5); 6% wt. (MS6); 7.5% wt. (MS7.5); 10% wt. (MS10); 15% wt. (MS15); and 20% wt. (MS20) as a partial substitute for cement;
- For nanosilica (NS), in a proportion of 0.75% wt. (composition noted as CB1.5); 1.5% wt. (CB3); and 2.25% wt. (CB4.5) as a partial substitute for cement; the notation was made taking into account the weight of the colloidal form of nanosilica (Cembinder8 product noted CB, ≈50% wt. NS content) in the mortars’ composition;
- For mixtures of microsilica (MS) and nanosilica (NS), in proportions of 3% wt. MS and 0.75% wt. NS (composition noted as MS3CB1.5); 4.5% wt. MS and 0.75% wt. NS (MS4.5CB1.5); 6% wt. MS and 0.75% wt. NS (MS6CB1.5); 3% wt. MS and 1.5% wt. NS (MS3CB3); 4.5% wt. MS and 1.5% wt. NS (MS4.5CB3); and 3% wt. MS and 2.25% wt. NS (MS3CB4.5) as a partial substitute for cement.

The progressive replacement of cement with micro- and/or nanosilica was performed with the aim of choosing the optimal compositional variant, from the point of view of the properties of interest, and finally to propose a mortar composition, with enhanced properties, to be used for the encapsulation of low-level radioactive waste (LLW). The variants for the micro- and nanosilica content were chosen taking into account the data presented in the specialized literature for similar systems made with natural aggregates [10,11,45,46,47,48,49].

Compositional characteristics, such as the weight ratio of water/(cement + silica), the weight ratio of recycled concrete aggregate/(cement + silica) and the weight content of micro- and nanosilica, are presented in Table 1 for all the compositions made, together with those of the reference mortar (only with ordinary Portland cement, OPC).

### 2.2. Experimental Conditions

**Figure 1:**
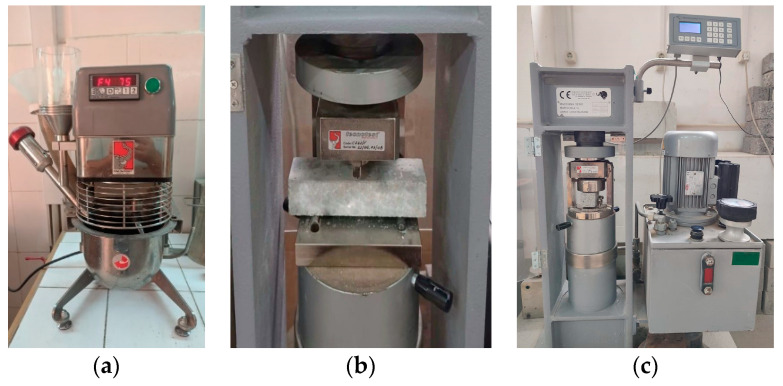
Images from the mixture homogenization (**a**), the flexural tests (**b**) and the compression tests (**c**) of the samples.

The homogenization of the mixture of raw materials was conducted with the help of a programmable mixer with forced mixing (Figure 1a), with a capacity of 5 L, according to EN 196-1 [50]. The additives were introduced into the mixing water.

Immediately after preparation (0 min), the flow time of a quantity of 1000 mL of mortar was determined, with the Marsh funnel, through the nozzle with a diameter of 10 mm. The test was repeated after 15 min, 30 min and 60 min to verify the homogeneity of the samples.

The amounts of water in the recipes were adjusted during the flow tests in order to fall within the limits of 25–35 s/1000 mL of mortar, while the content of the superplasticizer (1.5% wt. relative to cement) and viscosity modifier (0.5% wt. relative to cement) was kept constant for all compositions.

In order to obtain samples necessary for mechanical determinations, mortar prisms were cast, using steel molds with three compartments, with dimensions of 40 × 40 × 160 mm. After demolding, the samples were kept immersed in water for up to 7 or 28 days, at a temperature of about 20 °C.

Before being mechanically tested, the samples were weighed to determine the apparent density of the hardened mortar. For each experimental condition, the flexural strength was determined, on three samples, with a test press with a capacity of 15 kN and a loading speed of 50 ± 10 N/s, as shown in Figure 1b.

The compressive strength was obtained on the prism halves remaining from the flexural test (6 samples for each experimental condition); the test press had a capacity of 250 kN and a loading speed of 2400 ± 200 N/s (Figure 1c).

## 3. Results

### 3.1. Characterization of Cement, Micro- and Nanosilica

The elemental chemical composition (by X-ray fluorescence analysis) and the results of the Brunauer–Emmett–Teller (BET) surface area analysis for cement and microsilica (MS) are presented in Table 2 and Table 3. The high SiO_2_ content of microsilica and the high specific surface area are highlighted.

For the case of densified microsilica (MS) powder, the determined physical characteristics are presented in Table 4 and those for the granulometric distribution are presented in Table 5.

It stands out from the granulometric composition of the microsilica powder that the rest of the powder on the 90 µm sieve is approx. 80%, which is significantly higher than that corresponding to the cement used (R_0.09_ = 1.2%) or any other ordinary Portland cement [45]; this is due to the densification process. Consequently, the higher specific surface area of microsilica compared to that of cement is due to the increased porosity of densified microsilica and not to the size of the particles.

The average value obtained for the pH of Levasil CB8 colloidal nanosilica is 9.4 units and the density is 1.399 g/cm^3^.

**Figure 2:**
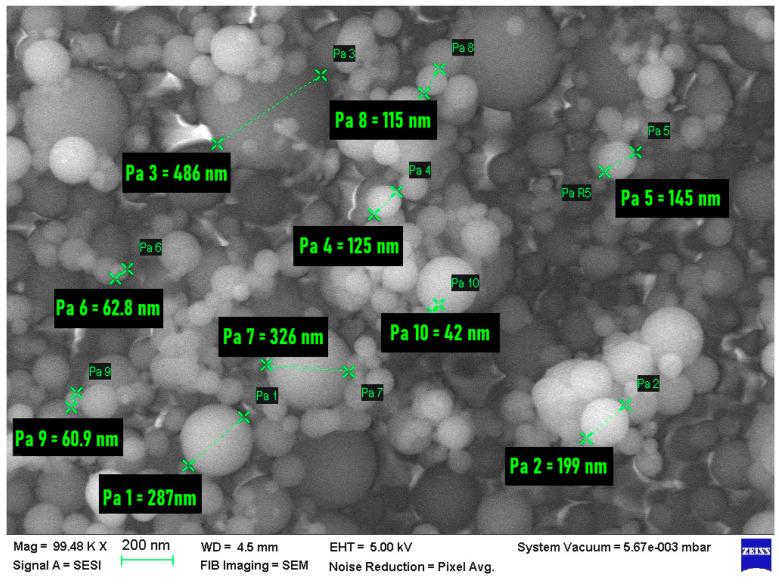
Micrograph of the MS sample, at 1 × 10^5^ magnification, highlighting the average particle sizes.

**Figure 3:**
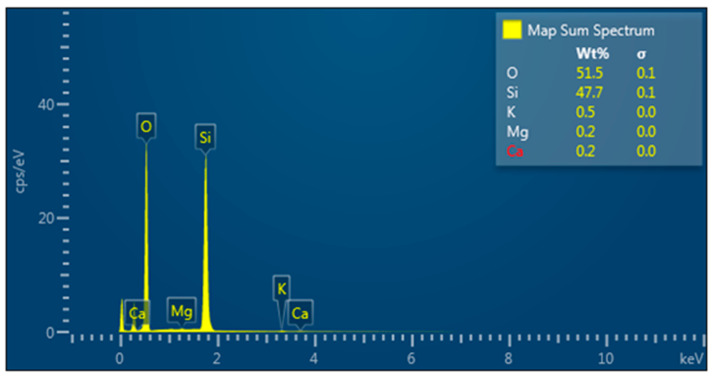
EDS spectra, with the quantitative identification of the constituent elements.

FESEM-FIB microstructural analysis, coupled with EDS, for densified microsilica (MS), highlighted the following:

- From the morphological point of view, the microsilica sample is made up of spherical or spheroidal-shaped particles, with dimensions between 40 nm and 500 nm, which are found in the form of agglomerations (conglomerates) (Figure 2);

- The chemical composition, determined punctually with the help of the EDS, showed the presence of SiO_2_ as the majority phase in MS and small concentrations of K, Mg and Ca oxides (Figure 3), results that are in agreement with those obtained by X-ray fluorescence spectrometry;
- The powder looks uniform from a compositional point of view, without the appearance of contamination or impurities.

While using the Chapelle method for determining the pozzolanic activity of micro- and nanosilica, an approximately double amount of Ca(OH)_2_ related to nanosilica resulted compared to that related to microsilica (Table 6); this fact is due to the SiO_2_ nanoparticles that have most of the atoms on the surface. This characteristic enhances their reactivity at the surface and is responsible for stronger chemical interactions at the interfaces [3,8].

### 3.2. The Compatibility test between Cement–Additive–Micro/Nanosilica

**Figure 4:**
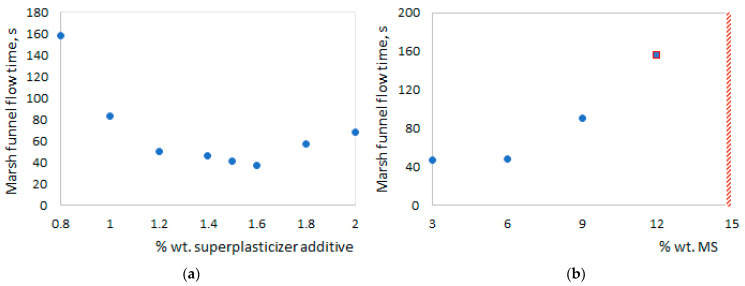
The flow time of the paste, in seconds, depending on the % wt. of the superplasticizer (**a**) and the % wt. of the MS (**b**).

The compatibility test between the unitary cement CEM I 42.5 R and the superplasticizer additive used shows an important increase in the fluidity of the standard cement paste (500 g cement and 170 mL water), evidenced by the reduction in the flow time through the nozzle of the Marsh funnel at a mass content of the additive included between 1.4 and 1.6% wt. relative to cement (Figure 4a).

The steric effect exerted by the layer of polycarboxylated ethers adsorbed on the surface of the hydrated phases of the cement leads to the dispersion of the cement particles and prevents their reagglomeration. Increasing the fluidity of mortars without increasing the water content is one of the objectives pursued to fulfill the technical criteria applied to LLW concrete waste; this also leads to an increase in the durability of cement systems. For this reason, for the subsequent determinations and the mortars made, a content of 1.5% wt. superplasticizer additive was kept constant, relative to cement.

The compatibility tests between the cement—the superplasticizer additive and micro/nanosilica showed an improvement in the cohesion of the paste manifested by the lack of segregation and the increase in the flow time with the increase in the replacement percentage of the cement with silica, regardless of whether the SiO_2_ particles were at the micro- or nanometric level. At 12% wt. MS, the flow was discontinuous (in drops), and over 15% wt. the MS paste no longer showed flow (the red hatched area) (Figure 4b). NS behaved similarly, but at lower weights; at 1.5% wt. NS, the paste flowed slowly, intermittently and in drops, and over 4.5% wt. the paste no longer flowed through the 10 mm nozzle of the Marsh funnel.

### 3.3. Characterization of Mortars

The flow tests of the fresh mortars, carried out straight after mixing (0 min.) with minimal amounts of water in the mixture of components, showed that they fit the technical criteria imposed (16–35 s/1000 mL mortar flow time). The same thing happened after 15, 30 and 60 min. of stationary storage of mortars, with the flow time increasing by only 2 to 7 s. The flow time values, in seconds, together with the fresh apparent density of the mortars, are presented in Table 7.

All mortars with micro- and nanosilica have very good homogeneity, they are cohesive and they do not present water bleeding. A single exception was observed in the case of the MS6 composition, which exhibited a light water separation of 2.5 mL of water per 1000 mL of mortar, compared to the control mortar (OPC) where the quantity was 4.7 mL water/1000 mL mortar. The higher the degree of replacement of cement with silica, the lower the values of the apparent density for mortars with micro- and nanosilica compared to the control mortar (OPC) are, because the density of cement is higher than that of silica.

**Figure 5:**
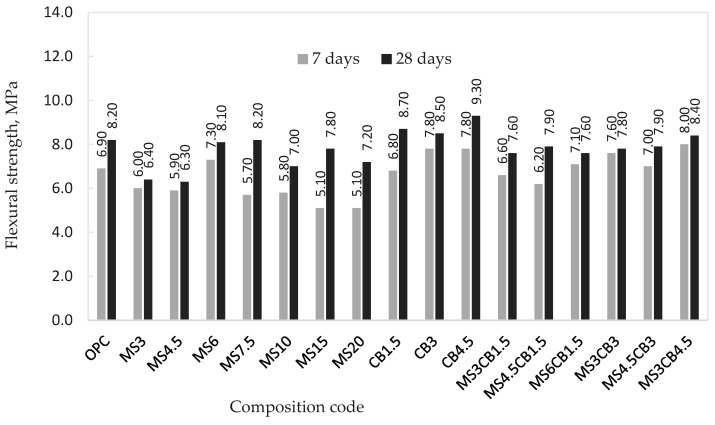
Flexural strength of mortar samples.

Figure 5 shows the flexural strength values, obtained at 7 and 28 days, for all investigated mortar compositions. The results, in MPa, are shown in comparison with the flexural strength value for the control mortar (without micro/nanosilica).

From the data presented, the following trends are highlighted:

- For the samples with microsilica, only the MS6 composition shows, at 7 days, higher values of flexural strength compared to the control composition sample (OPC);
- After 7 days, samples with nanosilica show, in general, higher flexural strength values compared to the control sample, a fact also evident in the case of mixed compositions with micro- and nanosilica, MS6CB1.5, MS3CB3, MS4.5CB3 and MS3CB4.5;
- At 28 days, the best results for flexural strength were obtained for samples with nanosilica, with the three compositions, CB1.5, CB3 and CB4.5, showing an increase in flexural strength of 6% to 13.4% greater than that corresponding to the control sample (OPC);
- Among the samples with mixed silica content (micro- and nanosilica), only the MS3CB4.5 composition shows a higher value, at 28 days, than the control composition.

**Figure 6:**
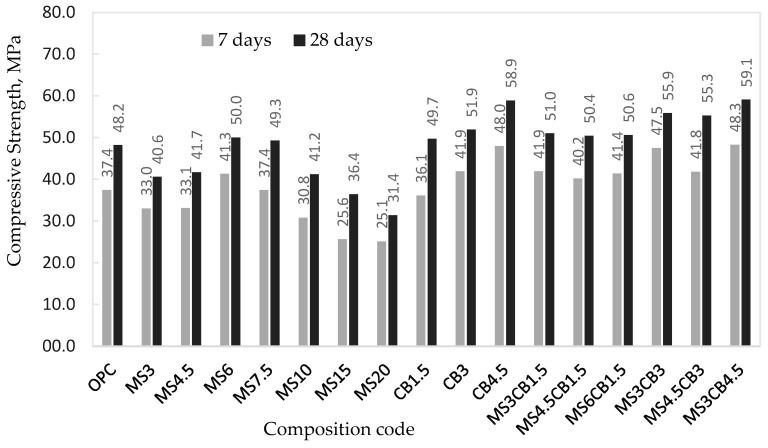
Compressive strength of mortar samples.

Figure 6 shows the compressive strength values, obtained at 7 and 28 days, for all investigated mortar compositions. The results, in MPa, are presented in comparison with the compressive strength value for the control mortar (without micro/nanosilica). Comparisons between the compressive strength values for mortars with recycled aggregates and similar mortars made with natural aggregates were presented in a previous study [27].

From the data presented, the following trends are highlighted:

- For compositions with microsilica, the compressive strength at 28 days shows the highest values, above that of the control sample (OPC), for samples with a content between 6 and 7.5% microsilica (MS6, MS7.5); for a content higher than 10% microsilica, the strength drops significantly. The behavior is similar to that found in mortars with natural aggregates where a maximum increase in mechanical strength is mentioned, in the specialized literature, for a cement replacement content of 7–9% wt. silica fume [38,40,46,47,49]. The use of densified microsilica makes the pozzolanic reaction with Ca(OH)_2_ take place more slowly; as a result, the compressive strengths for samples MS3 and MS4.5 show lower values than the control sample (OPC) but also compared to samples MS6 and MS7.5 at a close W/(C + S) ratio;
- In the case of samples with colloidal nanosilica, the compressive strengths (at 28 days) are superior to that of the control sample for all mortar compositions analyzed (CB1.5, CB3, CB4.5), with different percentages of nanosilica (0.75%, 1.5%, 2.25% wt.). Also, the compressive strength values increase significantly with the increase in nanosilica content; the sample with 2.25% wt. NS (CB4.5) shows the highest compressive strength (58.9 MPa). It shows a 22.2% increase in strength compared to the control sample (48.2 MPa) and 17.8% compared to the sample with microsilica with the best results, MS6 (50 MPa);
- If micro- and nanosilica are used simultaneously, a synergistic effect of silica is observed which makes the compressive strengths for those samples higher than those corresponding to samples with only nanosilica; thus, the compositions MS3CB1.5, MS4.5CB1.5 and MS6CB1.5 show strength values between 50.4 and 51 MPa (at 28 days) compared to the CB1.5 composition with 49.7 MPa; the MS3CB3 and MS4.5CB3 compositions have values in the range of 55.3–55.9 MPa compared to the CB3 composition with 51.9 MPa, and the MS3CB4.5 composition has the highest compressive strength, 59.1 MPa, compared to CB4.5 with 58.9 MPa. In conclusion, the greatest increase in compressive strength compared to the control mortar (OPC) is 23.5%, in the case of the MS3CB4.5 composition;
- For samples hardened at 28 days, the statistical analysis shows that 99.7% of the results have a standard deviation, 3σ, of ±22.5 MPa compared to the control sample value (OPC);
- The correlations highlighted for the hardening period of 28 days are generally preserved in the case of mortars hardening at 7 days.

**Figure 7:**
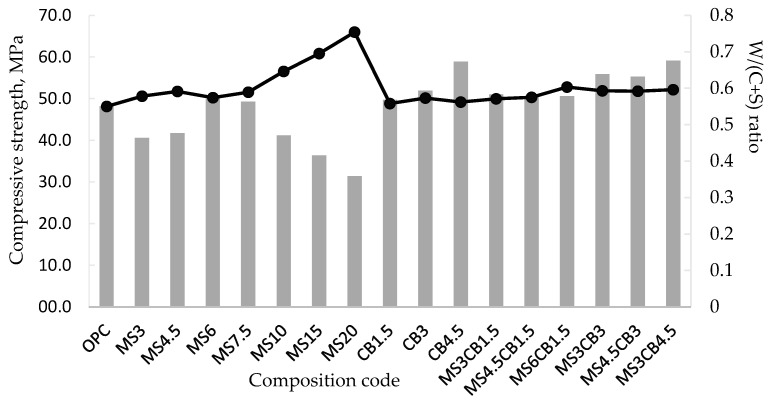
Correlation between compressive strength and W/(C + S) ratio.

The results of the compressive strength, at 28 days, must be analyzed in correlation with the ratio of water/(cement + silica) (W/C + S) used to obtain the respective mortar compositions (Figure 7). Increasing the proportion of microsilica leads to an increase in the W/(C + S) ratio, while maintaining the fluidity of the mortars (the Marsh flow time); the upward trend is limited in the case of compositions with 6% and 7.5% microsilica. Consequently, the compressive strength shows a maximum for the MS6 composition; for all other compositions, the resistance decreases with the increase in the microsilica content, due to the significant increase in the W/(C + S) ratio.

In the case of compositions with colloidal silica, the increase in the percentage of nanosilica occurs to a lesser extent than that of microsilica in MS compositions, which makes the increase in the W/(C + S) ratio lower for the same viscosity of the mortars. This is also evident for the mixed micro/nanosilica compositions, taking into account that the proportion of microsilica varies only between 3 and 6%. Consequently, the increase in compressive strength for compositions with colloidal silica is due to the following:

- The very large specific surface area of nanosilica correlated with nanosilica content;
- The synergistic effect of the micro- and nanosilica content in the mixed compositions.

Table 8 shows the values of the specific surface, the volume of the open porosity and the average diameter of the pores (BET analysis) for the hardened samples (at 7 days) with the best results in terms of compressive strength. A single composition was chosen for each category, namely the control mortar (OPC); mortars with microsilica—MS6; mortars with colloidal silica—CB4.5; and mortars with micro- and nanosilica—MS3CB4.5.

The results indicated the following:

- The adsorption–desorption isotherms of nitrogen obtained for the mortar samples hardened at 7 days are of type IV (according to the IUPAC classification), with a hysteresis curve characteristic of mesoporous solid materials;
- The specific BET surface area values for the samples with micro/nanosilica are between 15.89 and 21.33 m^2^/g, below the value of the control sample, 21.77 m^2^/g;
- The total pore volume has values between 0.954 × 10^−1^ and 1.118 × 10^−1^ cm^3^/g for samples with micro/nanosilica, compared to the value of the control sample, 1.332 × 10^−1^ cm^3^/g;
- The average pore diameter is 24.48 nm for the control sample and the samples with micro/nanosilica show smaller values between 20.98 and 24.02;
- All the values obtained show a reduction in the porosity of the samples due to the presence of micro/nanosilica. The increase in structural compactness leads to an increase in the mechanical resistance and, implicitly, in the durability of the cementitious compositions [10,11,12,14].

**Figure 8:**
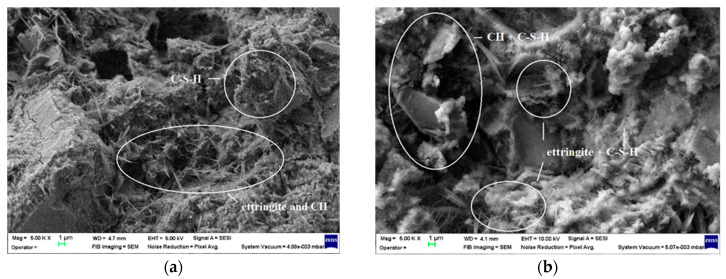
SEM images of the OPC mortar specimens: (**a**) after 7 days of curing, 5 × 10^3^ magnification; (**b**) after 28 days of curing, 5 × 10^3^ magnification.

SEM analyses were used to study the influence of microsilica (MS) and nanosilica (NS) on the microstructure of the OPC mortar. SEM images of the fracture of OPC, MS6, CB4.5 and MS3CB4.5 samples after 7 and 28 days of curing are shown in Figure 8. The fracture surface of the OPC sample is typical for a cement mortar, displaying a heterogeneous distribution of calcium silicate hydrate (C-S-H) and calcium hydroxide (CH) grains and needle-like ettringite crystals [2,5,46,48]. Initially, after 7 days of hardening, the ettringite crystals were already well outlined (Figure 8a), and at 28 days of hardening the increase in the degree of hydration of the cement led to an obvious increase in the proportion of C-S-H (Figure 8b).

**Figure 9:**
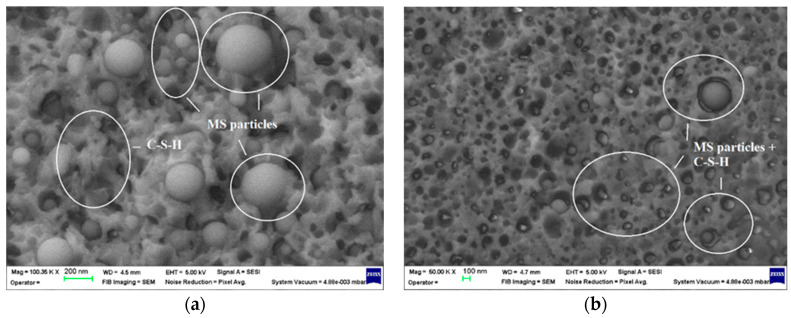
SEM images of the MS6 mortar specimens: (**a**) after 7 days of curing, 1 × 10^5^ magnification; (**b**) after 28 days of curing, 5 × 10^4^ magnification.

In the case of the MS6 samples, microsilica particles of different sizes, distributed arbitrarily throughout the hydrated cement products, are very evident (Figure 9a,b). Comparing the SEM images for the two hardening periods, we can observe the consumption of microsilica to a large extent after 28 days, especially for the smaller particles, following the reactions with calcium hydroxide. Also, the formation of additional amounts of calcium hydrosilicates around the microsilica particles after 28 days of hardening (Figure 9b), compared to the hardening period of only 7 days (Figure 9a), is clearly highlighted.

**Figure 10:**
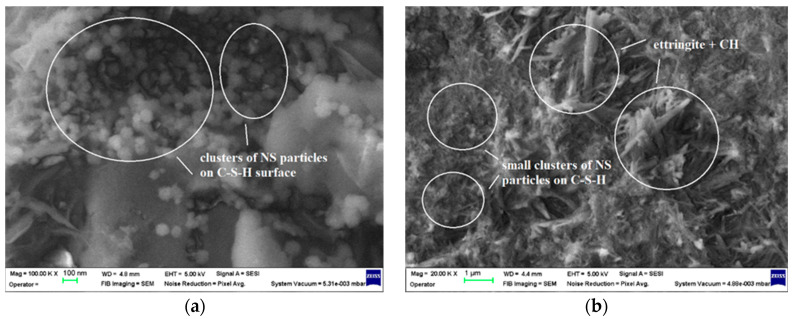
SEM images of the CB4.5 mortar specimens: (**a**) after 7 days of curing, 1 × 10^5^ magnification; (**b**) after 28 days of curing, 2 × 10^4^ magnification.

The SEM images of the fracture surfaces of CB4.5 samples after 7 and 28 days of curing reveal the presence of NS in the form of agglomerations of nanoparticles (clusters) on the surface of the cement hydration products. The shape of these clusters is still well defined after 7 days of hardening (Figure 10a), but after 28 days of hardening (Figure 10b) the clusters of NS have a smaller size and a shape that is harder to notice in the mass agglomeration of additionally formed C-S-H.

**Figure 11:**
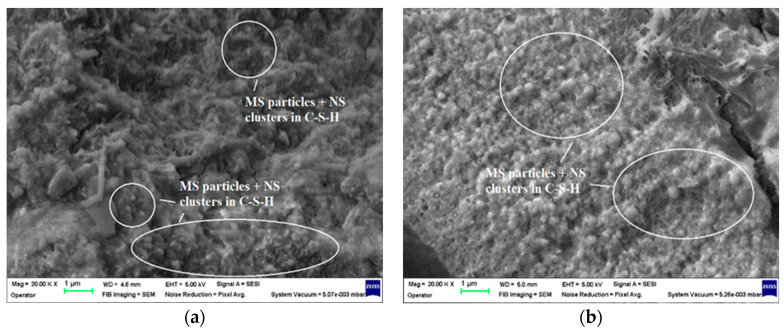
SEM images of the MS3CB4.5 mortar specimens: (**a**) after 7 days of curing, 2 × 10^4^ magnification; (**b**) after 28 days of curing, 2 × 10^4^ magnification.

In the case of the MS3CB4.5 mortar samples, the SEM images highlight important amounts of micro- and nanosilica arbitrarily dispersed among the hydration products of the cement. Both after 7 days of hardening (Figure 11a) and especially after 28 days of hardening (Figure 11b), condensed packing of cement hydration products can be observed, which indicates an intense reaction between micro/nanosilica and Ca(OH)_2_ and an increased compaction of the structure.

## 4. Discussion

The use of micro- and nanosilica in the cementitious compositions used for encapsulating radioactive waste in concrete has an obvious beneficial effect on both the fresh and hardened properties of the mortars made.

In the fresh state, the cohesiveness of the compositions is improved and the bleeding is limited, which reduces the risk of segregation of the cement mortars in the final storage barrels. This is more obvious in the case of the use of colloidal nanosilica, even at a lower percentage content than densified microsilica; the very small particles of amorphous SiO_2_ accelerate the hydration process of the mineralogical compounds of the cement. The use of nanosilica in a colloidal state also has the advantage of avoiding the tendency of SiO_2_ particles to agglomerate, which occurs in the case of using dry powder; it also avoids the excessive use of additives or water to disperse the particles. Thus, due to sodium stabilization of the surface of the SiO_2_ particles in the aqueous dispersion medium, colloidal nanosilica keeps a very large specific surface, and is more efficient than nanosilica in the form of dry powder.

From a structural point of view, due to the SiO_2_ particles under 50 nm in size and the very large specific surface area, the filler effect and the pozzolanic reaction [4,7,8,9,49] with Ca(OH)_2_ are more intense in the case of colloidal nanosilica than of densified microsilica (with the remainder on the sieve being 0.09 µm larger than the cement used). This is also highlighted by the SEM images, which reveal an increased densification of the structure, even at short hardening periods, when colloidal nanosilica is introduced into the composition of the mortars. Also, the acceleration of calcium silicate hydration by colloidal nanosilica also potentiates the pozzolanic reaction of densified microsilica, thus explaining the synergistic effect of the simultaneous use of micro- and nanosilica. In support of what has been stated are the higher values of the mechanical resistances for the compositions with micro- and nanosilica compared to those of the compositions with only colloidal nanosilica.

The slower pozzolanic reaction in the case of densified microsilica compared to colloidal nanosilica, also revealed by the SEM images, suggests the need to check the mechanical strength and, implicitly, the durability of the mortars obtained for periods longer than 28 days, as it is expected that the values of the properties of interest will be improved.

Further studies will focus on the evaluation of the retention capacity of radionuclides in the obtained cementoid systems by measuring the radiological activity of the leachate, using Cs^137^ and Co^60^ as radioactive isotopes [51,52,53].

## 5. Conclusions

The use of supplementary cementitious materials in mortars and concretes based on Portland cement has been and is still being studied for reasons of environmental protection and/or to increase the performance of such cementitious systems. Most of the research refers to systems made with natural aggregates and less to those containing recycled aggregates from concrete waste, it being known that the lower quality of the recycled aggregate leads to a decrease in the performance of the final mortars and concretes. However, there are special cementoid systems, such as mortars for encapsulating low-level radioactive waste, for which it has been shown in previous studies [27,51] that the use of recycled aggregates has significant benefits; it increases the degree of filling of the storage containers with radioactive waste, which reduces the storage volume in the warehouses.

The present study followed the use of micro- and nanosilica as hydraulically active admixtures to increase the compactness and durability of these special mortars made with recycled aggregates. The research program started from a mortar composition made with recycled aggregates that was optimized in a previous study [27].

The final goal is to increase the degree of fixation in the cement matrix of the radionuclides originating from the concrete waste resulting from the decommissioning of some nuclear installations.

A series of mortars were made with recycled concrete aggregate in which the cement was replaced, in different percentages, with microsilica, nanosilica and a mixture of the two, under the conditions of fulfilling the technical criteria applicable to such cementoid matrices that encapsulate low-level radioactive waste.

Based on the test results, the following conclusions are drawn:

- The microsilica used is a densified product with a high proportion of particles larger than those of cement, with the rest product on the 90 µm sieve being approx. 80%. For this reason, the pozzolanic reaction is delayed, and the compressive strength is higher than that of the control mortar only in the case of samples with 6% wt. MS and 7.5% wt. MS (an average increase of 3%). For the compositions with 10% wt. (MS10), 15% wt. (MS15) and 20% wt. (MS20) microsilica, the compressive strength decreases significantly compared to the control mortar due to the increase in the W/(C + S) ratio;
- The pozzolanic activity of colloidal nanosilica is clearly superior to densified microsilica, with all mortar compositions with nanosilica showing superior compressive strength to the control sample (OPC). The percentage increase in strength was higher as the proportion of nanosilica was higher; thus, the increase was 3.1% for CB1.5, 7.7% for CB3 and 22.2% for CB4.5;
- The compressive strength values for samples with mixed content (micro- and nanosilica), especially over 1.5% wt. nanosilica, highlight the emergence of a synergistic effect of the simultaneous use of micro- and nanosilica; the MS3CB4.5 composition shows the highest percentage increase in compressive strength compared to the control sample (23.5%);
- For the studied cementitious systems, the increase in flexural strength is manifested only in samples with nanosilica. The percentage increase is lower than in the case of compressive strength;
- The results of the mechanical tests for the compositions made are in close correlation with the rheological characteristics and the water/cementitious materials ratio;
- The increase in the structural compactness of mortars with nanosilica and a mixture of micro- and nanosilica is evidenced by the microstructural analysis, using a field emission scanning electron microscope with an integrated focused ion beam (FESEM-FIB) and a surface evaluation device by nitrogen adsorption (BET analysis).
- The formation of CSH following pozzolanic reactions takes place faster in the case of compositions with nanosilica compared to compositions with microsilica due to the larger specific surface area of nanosilica, something highlighted by the SEM images.

Even if, in general, the behavior of micro- and/or nanosilica additions in the mortars made is similar to that encountered in cementitious systems with natural aggregates, the compositional optimization is more difficult taking into account the technical criteria that have to be kept for such mortars and the inconveniences brought by the recycled aggregate (high porosity and permeability).

This study highlighted the beneficial influence of, in particular, nanosilica in colloidal form and the mixed addition of micro- and nanosilica on the properties of radiological encapsulation mortars. Considering the compositions achieved, the variant MS3CB4.5 can be chosen as the optimal composition proposed for used in the encapsulation of low-level radioactive waste.

## Figures and Tables

**Table 1:** The composition of mortars with microsilica and/or nanosilica.

Composition	Ratio W/(C + S)	Ratio RCA/(C + S)	MS wt.%	NS wt.%
OPC	0.550	1.3	0	0
MS3	0.578	1.3	3	0
MS4.5	0.591	1.3	4.5	0
MS6	0.574	1.3	6	0
MS7.5	0.589	1.3	7.5	0
MS10	0.646	1.3	10	0
MS15	0.695	1.3	15	0
MS20	0.754	1.3	20	0
CB1.5	0.558	1.3	0	0.75
CB3	0.573	1.3	0	1.5
CB4.5	0.562	1.3	0	2.25
MS3CB1.5	0.571	1.3	3	0.75
MS4.5CB1.5	0.575	1.3	4.5	0.75
MS6CB1.5	0.603	1.3	6	0.75
MS3CB3	0.593	1.3	3	1.5
MS4.5CB3	0.592	1.3	4.5	1.5
MS3CB4.5	0.596	1.3	3	2.25

**Table 2:** The oxide composition of cement and densified microsilica powder, in wt.%.

Sample	MgO	Al_2_O_3_	SiO_2_	CaO	SO_3_	K_2_O	TiO_2_	Fe_2_O_3_	MnO	ZnO
Cement	3.70	6.37	22.25	58.94	4.48	0.96	0.22	3.01	0.05	0.02
Microsilica	0.30	-	97.43	0.70	0.29	1.10	-	0.12	0.03	0.03

**Table 3:** BET analysis for cement and densified microsilica powder.

Sample	Specific Surface Area, m^2^/g	Total Volume of Open Pores, cm^3^/g	Average Pore Diameter, nm
Cement	1.034	1.281 × 10^−2^	49.56
Microsilica	17.88	3.621 × 10^−1^	81.00

**Table 4:** Physical properties of densified microsilica powder.

Property	Unit	Measured Value
Relative humidity	%	0.9
Powder retained on a 45 µm sieve	%	92.8
Loss on ignition, 975 °C	%	2.35
Absolute density	kg/m^3^	2174
Bulk density	kg/m^3^	684

**Table 5:** Granulometric distribution of densified microsilica powder.

Granulometric Sort, mm	wt.%
>1.0	1.2
0.500–1.0	1.0
0.250–0.500	44.0
0.125–0.250	35.5
0.09–0.125	0.2
0–0.09	20.1

**Table 6:** Chapelle method results.

Silica Type	v_1_ (mL sol HCl, 0.1 N)	mgCa(OH)_2/_1 g Silica
Densified microsilica	13.5	714
Colloidal nanosilica	7.6	1557

**Table 7:** Properties in fresh condition of mortars.

Composition	Flow Time (s), after				Density,
	0 min	15 min	30 min	60 min	kg/m^3^
OPC	27	29	31	33	2023
MS3	28	29	31	34	2012
MS4.5	25	28	31	35	2006
MS6	27	29	30	36	1991
MS7.5	26	28	29	31	1981
MS10	27	29	31	35	1974
MS15	26	27	28	30	1946
MS20	29	29	29	31	1928
CB1.5	28	29	29	32	2000
CB3	27	27	27	29	1994
CB4.5	26	29	30	33	2006
MS3CB1.5	28	30	31	34	1993
MS4.5CB1.5	27	31	35	38	2012
MS6CB1.5	25	27	29	38	1997
MS3CB3	26	27	28	30	2027
MS4.5CB3	25	26	27	31	2003
MS3CB4.5	25	26	29	32	2006

**Table 8:** The results of the BET analysis for hardened mortar samples.

Sample	Specific Surface Area, m^2^/g	Total Volume of Open Pores, cm^3^/g	Average Pore Diameter, nm
OPC	21.77	1.332 × 10^−1^	24.48
MS6	15.89	0.954 × 10^−1^	24.02
CB4.5	21.33	1.118 × 10^−1^	20.98
MS3CB4.5	20.28	1.084 × 10^−1^	21.38

## Data Availability

The data presented in this study are available on request from the corresponding author due to privacy restriction.
